# Nutritional status and vitamin A and zinc levels in patients with kala-azar in Piauí, Brazil

**DOI:** 10.1590/0037-8682-0800-2020

**Published:** 2021-09-06

**Authors:** Mísia Joyner de Sousa Dias Monteiro, Maria Nauside Pessoa da Silva, Adriana de Azevedo Paiva, Dilina do Nascimento Marreiro, Liania Alves Luzia, Gilberto Simeone Henriques, Patrícia Helen de Carvalho Rondó, Ingridi de Souza Sene, Ana Tárcila Alves de Almeida, Carlos Henrique Nery Costa, Dorcas Lamounier Costa

**Affiliations:** 1 Universidade Federal do Piauí, Mestrado em Ciências e Saúde, Teresina, PI, Brasil.; 2 Universidade Federal do Piauí, Programa de Pós-Graduação em Biotecnologia, Rede Nordeste de Biotecnologia, Teresina, PI, Brasil.; 3 Universidade Federal do Piauí, Departamento de Nutrição, Teresina, PI, Brasil.; 4 Universidade de São Paulo, Laboratório de Micronutrientes, São Paulo, SP, Brasil.; 5 Universidade Federal de Minas Gerais, Escola de Enfermagem, Laboratório Experimental de Nutrição, Belo Horizonte, MG, Brasil.; 6 Universidade de São Paulo, Departamento de Nutrição, Escola de Saúde Pública, São Paulo, SP, Brasil.; 7 Universidade Federal do Piauí, Laboratório de Pesquisas em Leishmanioses, Teresina, PI, Brasil.; 8 Laboratório de Anatomia Patológica e Biologia Molecular, Teresina, PI, Brasil.; 9 Universidade Federal do Piauí, Curso de Farmácia, Teresina, PI, Brasil.; 10 Centro de Inteligência para Agravos Tropicais Emergentes e Negligenciados, Teresina, PI, Brasil.; 11 Universidade Federal do Piauí, Departamento de Medicina Comunitária, Teresina, PI, Brasil.; 12 Universidade Federal do Piauí, Departamento Materno-Infantil, Teresina, PI, Brasil.

**Keywords:** Leishmaniasis, Visceral, Kala-azar, Malnutrition, Vitamin A, Zinc

## Abstract

**INTRODUCTION:**

Malnutrition and kala-azar (or visceral leishmaniasis) are significant public health problems in different parts of the world. Immunity and susceptibility to infectious and parasitic diseases are directly linked to the host’s nutritional state, but little is known about the interaction between nutrition and kala-azar. This study aimed to evaluate nutritional status with kala-azar and correlate these findings with the clinical and laboratory manifestations of the disease, and zinc and retinol levels.

**METHODS:**

This was a cross-sectional study of 139 patients with kala-azar. Nutritional status classification was performed according to international recommendations. Parametric or nonparametric tests were applied whenever indicated in a two-sided test with a 5% significance level.

**RESULTS:**

Weight loss and malnutrition were more frequent in adults. Body mass index-for-age, fat area of the arm, and upper arm muscle area were significantly associated with probability of death. The presence of human immunodeficiency virus, hepatomegaly, and splenomegaly was correlated with nutritional assessment. Blood leukocyte and lymphocyte, serum creatine, and vitamin A levels were significantly higher in adult men. Vitamin A levels were highly associated with the level of hemoglobin and C-reactive protein (CRP) in multivariate analysis. All patients had reduced plasma zinc levels, but this finding had no association with the outcome variables.

**CONCLUSIONS:**

Malnutrition was correlated with severe disease and was more prevalent in older people with kala-azar. Vitamin A deficiency was associated with hemoglobin and CRP. Zinc levels were reduced in patients with kala-azar.

## INTRODUCTION

Kala-azar or visceral leishmaniasis (VL) is caused by *Leishmania infantum* protozoa in the Americas and is transmitted by infected female phlebotomine *Lutzomyia.* The signs and symptoms of VL include fever, hepatosplenomegaly, weight loss, and pancytopenia in most patients. If not treated, this disease can be lethal. The factors associated with a higher risk of death are the presence of anemia, jaundice, edema, bleeding disorders, bacterial infection, and human immunodeficiency virus (HIV) coinfection^1^ ^-^ ^3^. Approximately half of kala-azar patients in Brazil are children under the age of five^4^. Death is usually associated with systemic inflammation, bleeding, and bacterial infections. Immunity and susceptibility to infections and parasitic diseases are correlated with nutritional status. 

Malnutrition and infection are intrinsically linked, and this interaction results in the worsening of both. The role of nutrition in the prevention of disease and the reduction of morbidity and mortality is evident, particularly in regions with a high prevalence of infectious diseases, such as tuberculosis, leishmaniasis, HIV, and malaria^5^. 

The World Health Organization (WHO) and the World Bank Group estimate that 462 million people are underweight and that 1.9 billion adults (>18 years of age) are overweight/obese. Furthermore, 52 million children (<5 years of age) are wasted, with ~17 million severely wasted, 155 million stunted, and 41 million overweight^6^. According to estimates, there are 0.2 to 0.4 million VL cases with an overall case-fatality rate of 10%^7^. 

Malnutrition increases susceptibility to infections, such as leishmaniasis, and determines the severity of illness by several etiologic pathways in cases of under- and overnutrition: immune dysfunction, decreased epithelial integrity, altered metabolic rate, chronic inflammation, and altered dietary absorption. The interactions between nutritional status and infectious disease is complex because it involves interactions between the host, pathogens, and the environment^8^.

Although malnutrition has been associated with a higher probability of infection by *Leishmania spp.*, the mechanisms involved in the relationship between nutrition and human VL evolution are poorly explained^9^. It is unclear whether malnutrition is a risk factor for kala-azar or a consequence of the protracted consumption process^9^ ^-^ ^12^.

Selective or combined deficiencies of micronutrients or trace elements can contribute to worsening and delayed recovery from infectious and chronic diseases^13^ ^,^ ^14^. Vitamin A (VitA) has health-promoting and regulatory activities in both the innate and adaptive immune systems; therefore, it can improve immune function and enhance defense against many infectious diseases^15^ ^,^ ^16^. VitA supplementation has been shown to prevent respiratory and diarrheal deaths in many countries^17^. Serum retinol levels are lower in individuals with kala-azar^18^ ^,^ ^19^, but the role of the immune system in the evolution of the disease is still unclear. 

Several minerals are associated with an impaired immune system. Zinc plays a fundamental role in the production or biological activity of several interleukins, which, in turn, influences the development and functions of T and B lymphocytes, macrophages, and NK cells. Helper T cells Th1 and Th2, which promote resistance to infections, are also affected by reduced zinc levels^20^ ^,^ ^21^. Additionally, studies have shown that serum zinc levels are lower in people with kala-azar^22^. 

The objective of this study was to assess the nutritional status and VitA and zinc levels of patients with kala-azar and to correlate it with the clinical presentation of the disease.

## METHODS

### Patients

This study was conducted at the Natan Portella Tropical Diseases Institute in Teresina, Piauí, Brazil, from June 2018 to August 2019. Individuals of all ages and both sexes who presented with fever, splenomegaly, weight loss, or paleness and had previously undergone bone marrow puncture were included. Anamnesis and physical examinations were performed during the first 24 hours of hospitalization. The liver and spleen were measured by palpation and percussion during physical examination. Kala-azar was confirmed by a positive parasitic test (via microscopy or culture of *Leishmania sp.* in a Novy-McNeal-Nicolle medium or via polymerase chain reaction). An immunochromatographic test was conducted when the first tests could not be performed, and the diagnostic suspicion was high.

### Nutritional evaluation

Anthropometry was conducted by following the protocol of the Laboratory for Nutritional Evaluation of Populations^23^ and by using data from the National Health and Nutrition Examination Survey (CDC, 2017)^24^. The percentage of body weight loss during illness was calculated if information on weight at the onset of symptoms was available. Weight-for-age, height-for-age, body mass index (BMI), and respective percentiles and Z-scores were calculated using Anthro and AnthroPlus software (WHO, 2009). The muscular and fat areas of the arm were determined according to the proposal of Frisancho^25^. 

### Laboratory tests

Laboratory tests included blood count (laser flow cytometry), erythrocyte sedimentation rate (Westergreen method), aminotransferase dosage (Technicon automation method), bilirubin (colorimetric method), creatinine (Jaffé reaction), urea (Berthelot urase), alkaline phosphatase (Bowers & McComb), albumin and globulin (bromocresol green), C-reactive protein (CRP) (nephelometry), summary test (Uri-test 11), and urine culture. The laboratory reference value (accreditation by the General Coordination for Accreditation and the Institute National Metrology, Quality, and Technology [INMETRO, Brazil]) served as a reference for the laboratory results. Screening for HIV was part of the investigation of suspected kala-azar patients. Risk of death was calculated using KalaCal^®^ software^1^. VitA and serum and erythrocyte zinc measurements were performed as proxies for micronutrient malnutrition and followed published protocols^26^. 

### Retinol measurement

Serum retinol was measured using a high-performance liquid chromatography LC-20AT model equipped with an SIL- 20AC automatic injector, a CBM-20A system controller, and an SPD-M20A controller diode array detector (Shimadzu Inc. Tokyo, Japan). We defined “VitA deficiency” as VitA levels below 0.35 µmol/L, “low VitA levels” as between 0.35 and 0.70 µmol/L, and “sufficient VitA levels” as below 0.70 µmol/L^27^. 

### Zinc measurement

Zinc levels were analyzed by an inductive plasma spectrometer and optic emission spectrometry with an axial view configuration and a V-Groove nebulizer (720 ICP/OES, Varian Idc., California, USA). The spectral line used for zinc measurement was 213.856 nm. Samples of certified reference material and Seronorm^TM^ Oligoelement Serum L-1 and L-2 (Billingstad, Norway) were used to validate the analytical measurements. The spectral line used for zinc measurement was 213.856 nm. Samples of certified reference material and Seronorm^TM^ Oligoelement Serum L-1 and L-2 (Bill, instead, Norway) were used to validate the analytical measurements. The cutoff points for plasma zinc adequacy and erythrocyte zinc adequacy were 75 to 110 μg/dL and 40 to 44 µg/g Hb, respectively^28^ ^,^ ^29^.

### Ethical board approval

The Research Ethics Committee of the Federal University of Piauí approved the study (No. 2.445.702). Each participant or his legal representative signed an informed consent form.

### Statistical analysis

Nutritional analysis followed the definitions of the WHO^30^ ^,^ ^31^ and Health Ministry/System for Food and Nutritional Vigilance^32^. Categorical variables were analyzed using proportions. Quantitative data are presented as means, medians, confidence intervals (CIs), or interquartile intervals. Percentiles Z-scores and dispersion are presented as standard deviations of minimum-maximum intervals. The association of categorical variables was evaluated using a chi-square test and Fisher’s exact tests. The correlation between normally distributed variables was evaluated using the Pearson correlation coefficient or Spearman correlation test when the variables were not normally distributed. A t-test was performed to compare the means of normally distributed continuous variables, and the Wilcoxon rank-sum test was used to compare nonnormally distributed continuous variables. The VitA deficiency odds ratios for clinical and laboratory data were calculated using univariate and logistic multivariate analyses. The normality of numeric variables was visualized in dispersion graphs and evaluated using kurtosis and skewness tests. A 5% significance level for two-sided tests was considered in all analyses of VitA deficiency between the clinical and laboratory variables

## RESULTS

A total of 139 individuals were recruited: 102 (73.4%) were male, and 37 (26.6%) were female (*p* < 0.001). Age distribution was bimodal with a mean of 4.3 years (95% CI 3.3-5.4) in individuals under 18 years old and 39.0 years (95% CI 35.9-42.1) among individuals aged 18 years old or older. One hundred eight participants (77.7%) were from Piauí, 29 (20.9%) were from Maranhão, and 2 (1.4%) were from Bahia. Ninety-nine individuals (71.2%) lived in urban areas, and 40 (28.8%) lived in rural areas (*p* < 0.001). Seven (5.04%) patients died during hospitalization. Ninety-seven participants (69.8%) were diagnosed by parasitological diagnosis (microscopy or culture), and 42 (30.2%) were diagnosed by rapid immunochromatographic test (rapid test). A confirmatory molecular test revealed that 13 of 42 patients were positive; thus, 12 of 42 patients were diagnosed only by rapid testing. The mean time between the onset of symptoms until the diagnosis was 30.5 days (95% CI 24.9-36.2). Treatment for kala-azar was performed with pentavalent antimonial in 68 participants (48.9%) and with liposomal amphotericin in 71 participants (51.1%). 

The main signs and symptoms identified at hospital admission were fever, pallor, apathy, fatigue, and chills. The most frequent laboratory changes were anemia and leukopenia. Table 1 presents the demographic and clinical characteristics of the study population.

**Table:** 

Characteristic	n	%
**Sex**		
Male	102	73.4
Female	37	26.6
**Age (m, 95%CI)** ^1^		
< 18 y.o.	4.3	3.3-5.4
≥ 18 y.o.	39.0	35.9-42.1
**Origin**		
Urban	99	71.2
Rural	40	28.8
**Diagnostic method**		
Parasitological	97	69.8
Molecular	30	21.6
Immunological	12	8.6
Duration of illness (days)^2^(m, 95%CI)	30.5	24.9-36.2
**Signs and symptoms**		
Fever	133	95.7
Anemia	133	95.7
Splenomegaly	135	97.1
Thrombocytopenia	99	71.2
Neutrophils < 1000 cel/mm^3^	60	43.1
CRP^3^ >10 mg/dL	133	95.7
CRP > 50 mg/dL	81	60.9
**Risk of death** ^4^		
> 0.10	34	26.5
> 0.30	8	5.8

^1^age (mean, 95% confidence interval); ^2^Days from first symptoms to diagnosis; ^3^C-reactive protein; ^4^Risk of death calculated by KalaCal^®^ software.

### Nutritional analysis

Nutritional assessment according to age group (children under 5 years old, children between 5 and 10 years old, children 10 to 20 years old, adults, and elderly) is presented in the supplemental material (Supplemental Tables 1 to 4). Children and adolescents had a higher prevalence of malnutrition when assessed by weight-for-age than by height and BMI-for-age. Adults and the elderly had a BMI below normal limits and a higher prevalence of malnutrition than younger patients. Supplemental Figure 1 shows the graphs of weight-for-age, height-for-age, and BMI-for-age in children and adolescents, with the curves of weight-for-age and height-for-age shifted to the left.

One hundred and five patients (77.0%) reported their body weight before getting sick, and 82 (78.1%) had weight loss. There was a positive correlation between the percentage of weight loss and age (r = 0.29; *p* = 0.003), thus indicating that adults lost more weight than children.

Given that the population in this study comprised individuals from early childhood to old age, it was necessary to adopt a unified index to compare nutritional data for all ages. The BMI was adapted in three categories in each age group to define malnutrition, eutrophy, and overweight or obesity. After applying this interpretation to the nutritional diagnosis, we found that 23 participants (16.79%) were malnourished, 93 (67.88%) were eutrophic, and 21 (15.33%) had weights above the eutrophic limits (Supplemental Table 5). 

The mean age of the female participants was higher than that of the male participants (*p* = 0.007). The mean brachial circumference and skinfold were significantly lower in women, except for the subscapular fold and brachial fat percentage (Table 2). Data on brachial circumference and skinfolds were missing in some participants because of their refusal to undergo measurements. A sensitivity analysis was then performed to verify whether the loss of information would have been different. There was no difference in sex, main signs and symptoms, and laboratory tests between the groups with and without this measurement. Thus, the analysis was considered viable. Brachial circumference was the most sensitive parameter to detect malnutrition, with 83 participants (66.9%) below the 5th percentile. 

**TABLE 2:** Anthropometric measurements according to the sex of 139 individuals with kala-azar.

Variables	Male		Female		Total		
	mean	95%CI	mean	95%CI	mean	95%CI	*p-value*
Age (years)	24.0	20.0-28.0	13.9	8.5-19.3	21.3	18.0- 25.0	0.007
Age < 14	4.5	3.1-5.9	4.0	2.3-5.8	4.35	3.3-5.4	0.66
Age ≥ 14	39.4	35.8-42.9	37.2	32.2-42.1	39.0	35.9-42.1	0.38
BMI (Kg/m^2^)	19.2	18.4-20.1	17.1	15.9-18.4	18.6	17.9-19.4	0.01
BC (cm)	20.6	19.4-21.8	17.5	15.5-19.5	19.8	18.8-20.9	0.01
TSF (cm)	7.4	6.3-8.5	10.0	7.7-12.2	8.0	7.0-9.0	0.03
SSF (cm)	7.9	7.0-8.6	8.4	5.7-11.1	7.9	7.1-8.8	0.57
BMC (cm)	19.5	18.4-20.6	15.2	13.5-16.9	18.5	17.5-19.5	0.0001
BA (cm^2^)	36.3	32.2-40.4	26.7	20.0- 33.3	33.9	30.4-37.4	0.02
BFA (cm^2^)	7.4	6.2 -8.8	9.5	6.2-12.7	8.0	6.7 -9.2	0.18
% BFA	18.8	17.1 -20.5	29.7	26.5-32.9	21.5	19.8 -23.2	<0.0001

Student t-test. **BC:** Brachial circumference; **TSF:** Triceps skinfold; **SSF:** Subscapular skinfold; **BMC:** Brachial muscle circumference; **BA:** Brachial area; **BFA:** Brachial fat area; **% BFA:** Percentage of brachial fat area; **CI:** confidence interval; **BMI:** body mass index.

Table 3 shows the correlation analysis of the BMI classification according to age, upper arm muscle, and arm fat area with clinical and laboratory data. A positive correlation was observed between nutritional indicators and percentage of body weight lost during the disease, neutrophil and serum creatinine levels, and splenomegaly. Nutritional status was negatively correlated with hepatomegaly, leukocyte count, and lymphocyte count. 

**TABLE 3:** Correlation of BMI for age, upper arm muscle area, and upper arm fat area with clinical and laboratory variables.

Parameter	^1^ **BMI for age**		Upper arm muscle area		Upper arm fat area	
	r	***p-*value**	r	***p-*value**	r	***p-*value**
						
^2^weight loss (grams)	0.16	0.09	-0.22	0.04	0.03	0.73
Spleen extension (cm)	0.14	0.16	-0.24	0.03	-0.21	0.03
Liver extension (cm)	- 0.02	0.80	-0.04	0.72	-0.33	0.005
Leukocytes (cels/mm^3^)	- 0.23	0.006	-0.30	0.0008	0.20	0.04
Neutrophils (cels/mm^3^)	0.17	0.05	0.05	0.53	0.17	0.10
Lymphocytes (cels/mm^3^)	-0.17	0.04	-0.41	<0.0001	-0.31	0.001
Platlets (cels/mm^3^)	0.01	0.90	0.06	0.46	0.04	0.67
^3^CRP (mg/L)	0.11	0.22	0.21	0.04	0.19	0.06
Serum creatinine	0.24	0.02	0.67	<0.0001	0.30	0.01
Erythrocyte zinc (mg/dL)	-0.01	0.86	-0.01	0.99	-0.06	0.54

Pearson test. ^1^ **BMI:** body mass index; ^2^Percentage of weight lost during illness; ^3^C-reactive protein.

Men had a higher BMI, larger arm muscle area (*p* = 0.002), and larger upper arm muscle area (*p* = 0.01) than women, but there was no correlation between fat area and sex. There was a correlation between BMI adjusted for age (*p* = 0.02), muscle mass of the arm (*p* = 0.0001), and fat area of the arms (*p* = 0.002) when the groups were compared in terms of lethal outcome and probability of death. The presence of HIV infection, bacterial infection, or bleeding was not correlated with anthropometric variables.

### HIV-coinfected patients

Seventeen patients (12.2%) were coinfected with HIV-*Leishmania*. One HIV-coinfected patient was a 58-year-old malnourished male who had a prolonged febrile disease and died during VL treatment. The 12 remaining HIV-coinfected patients (92.3%) were adult males aged 25-58 years old. Malnutrition was correlated with HIV coinfection (*p* = 0.01), but there was no correlation when the analysis was controlled for age (*p* = 0.54). 

### Vitamin A

The mean level of VitA was 1.00 ?mol/L (95% CI 0.89-1.11). Considering the cutoff point of 0.75 ?mol/L, which includes individuals with deficient and low levels, 67 (48.2%) and 72 (51.8%) individuals were classified as sufficient and insufficient, respectively. Table 4 presents the association between VitA levels and the relevant clinical and laboratory variables. VitA levels were negatively associated with high levels of CPR (*p* = 0.002) and positively associated with hemoglobin levels below 9 mg/mL (*p* = 0.004).

**TABLE 4:** Mean levels of vitamin A, and association of vitamin A deficiency with clinical and laboratory variables.

	Serum				^4^ **Vitamin A deficiency**			
	Vitamin A				Univariate analysis		Multivariate analysis	
	n=138	mean (mmol / L)	95% CI	^1^ ***p*-value**	Odds ratio	^2^ ***p*-value**	Odds ratio	^3^ ***p*-value**
**Sex**								
Male	102	1.11	0.92-1.31	0.04	0.46	0.05	0.34	0.26
Female	36	0.72	0.51-0.93					
**Age**								
< 18 y.o.	71	0.88	0.69-1.09	0.11	1.60	0.17	2.15	0.38
≥ 18 y.o.	67	0.97	0.90-1.38					
**HIV coinfection**								
Absent	121	1.02	0.85-1.20	0.78	2.95	0.20	-	-
Present	17	0.96	0.62-1.30					
**Hemoglobin (mg/dL)**								
< 9	38	0.66	0.45-0.87	0.007	1.35	0.002	6.58	0.004
≥ 9	101	1.15	0.95-1.35					
^5^ **CRP (mg/L)**								
≥50	103	0.91	0.70-1.12	0.07	0.49	0.04	0.12	0.008
<50	29	1.20	0.95-1.46					
**Albumin (g/dL)**								
< 2.5	29	0.89	0.20-1.83	0.54	2.68	0.11	3.03	0.16
≥ 2.5	25	1.47	0.90-1.58					

^1^Student t- test; ^2^Chi-square test; ^3^Logistic regression. ^4^Vitamin A level < 75 μmol / L; ^5^C-reactive protein; **CI:** confidence interval.

### Zinc

Serum zinc levels were measured in 138 participants. The expected serum zinc values would be above 59 µg/dL for women and above 61 µg/dL for men. Zinc levels ranged from 30.9 to 44.9 µg/dL and were below the minimum limit of normality in all individuals. The mean erythrocyte zinc level was 38.14 (95% CI 37.7-38.5), and the mean plasma zinc level was 57.1% (95% CI 56.4-57.8). The minimum and maximum values were 48.4 and 66.8, respectively; therefore, all participants had plasma zinc levels below the minimum limit value. Erythrocyte zinc levels were statistically higher in HIV-coinfected patients than in noncoinfected ones (39.1 µg/dL versus 38.0 µg/dL), although all measurements were below the minimum values. Likewise, plasma zinc levels were also statically higher in coinfected patients than in noncoinfected ones (57.3 µg/dL versus 56.1 µg/dL). Erythrocyte zinc was statistically associated with serum urea (r = -0.24, *p* = 0.02), but it was not associated with other clinical, laboratory, demographic, housing, and sanitation variables. 

## DISCUSSION

Malnutrition, which is often present in individuals with kala-azar, is poorly understood. The population of this study was similar to the literature descriptions, with both sexes equally represented from childhood to adolescence and with men’s predominance in adulthood^1^ ^,^ ^33^ ^-^ ^36^. The age distribution was bimodal, with a higher mortality rate in young children and older people; this finding is possibly correlated with testosterone levels in adult men. Sex hormones, such as androgens, estrogens, and progestins can interact directly with the immune system and affect immune responses. Macrophages and lymphocytes, which are the two main types of cells involved in the outcome of leishmaniasis, both have receptors for sex hormones^37^. There was a higher frequency of kala-azar in participants residing in urban areas (99 [71.2%]) than in those living in rural areas (40 [28.8%]). This pattern of urbanization has been verified for at least four decades^38^ ^-^ ^40^. The severity of the disease can be inferred by the proportion of participants who presented with severe anemia, leukopenia, and thrombocytopenia, as described by several authors^1^ ^,^ ^12^ ^,^ ^35^ ^,^ ^36^ ^,^ ^41^ ^,^ ^42^. The levels of CRP were above 10 mg/dL in 95.7% of the participants, thus indicating acute inflammation; this result is similar to that of other studies^18^ ^,^ ^41^. The time elapsed from the onset of symptoms to diagnosis in individuals with VL in Brazil in the last few decades or in rural areas before the emergence of urban epidemics in Brazil was significantly less than that in the Old World^36^ ^,^ ^43^ ^,^ ^44^. Kala-azar in the Americas evolves into a severe disease that requires hospitalization and diagnosis sooner than in the Old World, and the time it takes for chronic protein-calorie malnutrition to occur is insufficient in the same proportion. The duration of symptoms until diagnosis was 30.5 days, whereas the mean time of the nonurban VL varied from a few weeks to many months^45^. 

Proportional weight loss in this study correlated positively with the individual’s age even when corrected by body weight before the illness, contrary to the literature, which shows greater vulnerability to nutritional deterioration in children^36^ ^,^ ^42^ ^,^ ^43^. There was a high prevalence of malnutrition in all age groups, as reported by other authors^18^ ^,^ ^43^. In addition, a higher prevalence has been reported in the Old World^46^ ^,^ ^47^. The evaluation of nutritional status on the basis of arm circumference, which is a parameter that can serve as an index of fat reserve and muscle mass, showed that 66.9% of people with kala-azar were malnourished^18^. In addition, by measuring the triceps skinfold, which allows for the identification of subcutaneous fat, 46% of individuals with kala-azar were malnourished or at risk of malnutrition. The analysis of the association between the various nutritional parameters and the clinical and laboratory variables was very similar when the BMI, muscular area, or fat area of the arm was considered, thus showing that malnutrition in patients with kala-azar involves both muscle loss and fat loss. 

HIV coinfection was statistically associated with few outcome variables, such as BMI and VitA levels, but did not have clinical relevance. Furthermore, this association was not verified by multivariate analysis. 

The association of serum VitA levels with age, sex, HIV coinfection, and disease severity was confounded by BMI, thus highlighting the great value of nutrition in this severe disease. These findings follow published studies that found that most kala-azar patients had low serum VitA levels^18^ ^,^ ^19^ ^,^ ^48^. In the current study, there was a moderate-to-strong correlation between VitA levels and age in years. This result is in line with an analysis of healthy children aged 6-13 years in China^49^. Breast milk remains an essential source of VitA in breastfed children from 7 to 12 months, but the national VitA supplementation program of the Ministry of Health of Brazil recommends that children from 6 to 59 months old receive preventive megadose of VitA, which may have interfered with the result^50^. 

VitA levels were significantly higher in men than in women in adulthood. However, when VitA sufficiency status was compared in each stratum, the difference only existed between men and women aged between 18 and 50 years, with 50% of women at this age having low levels of retinol. Weight was associated with VitA levels in women of reproductive age. The data in this study are similar to those of a clinical trial in which 40% of postpartum women had low VitA status and more than one-tenth had evidence of VitA deficiency^51^. Low VitA levels were associated with a probability of death greater than 0.10, but there was no association when the cutoff point of mortality was 0.3. Although it is impossible to infer causality in this study, the association between high CRP levels with low hemoglobin and CRP and VitA levels suggests that inflammation secondary to acute phase response, which is present in individuals with VL^52^, can partially explain anemia and VitA deficiency.

All individuals with kala-azar had serum zinc levels below the critical limit. The low levels of zinc found in this study can partly be attributed to the parasite-triggered inflammatory process and the host’s inability to deal with the infection mainly because of decreased cytokines and enzymes^53^. Although plasma zinc levels were higher in HIV-coinfected patients, all values were below the low limit of normality and were also very close. Although it is not possible to state whether this deficiency was a consequence of the prolonged consumptive state or a risk factor for disease progression, zinc supplementation may be beneficial for the recovery of these individuals. This finding is in line with those described in the literature^21^ ^,^ ^22^ ^,^ ^54^. Carbone et al.^55^ showed that children with kala-azar who received zinc supplementation during amphotericin B or pentavalent antimonial treatment showed faster clinical improvement than the group that did not receive zinc supplementation. However, a recent systematic review did not find any study that assessed the effects of oral nutritional supplements in patients with VL treated with antileishmanial drug therapy^56^.

This study showed that a significant proportion of kala-azar patients were malnourished and had lower VitA and zinc levels than the general population. Malnutrition was more prevalent in older people and was associated with severe disease in all age groups. Low levels of VitA were associated with nutrition and disease severity. Although zinc was not associated with the disease presentation and severity, it was reduced in all individuals with kala-azar. Finally, these findings led us to speculate whether VitA and zinc replacement may be beneficial as an adjuvant therapy for kala-azar.
