# Supplementary material for: Nutritional status and vitamin A and zinc levels in patients with kala-azar in Piauí, Brazil
Source: Rev Soc Bras Med Trop. 2021 Sep 6;54:e0800-2020. doi: 10.1590/0037-8682-0800-2020 (PMC8437443; doi:10.1590/0037-8682-0800-2020)
Supplement: Supplementary file 1 [file 1678-9849-rsbmt-54-e0800-2020-supp1.pdf]

SUPPLEMENTAL TABLE 1: Nutritional assessment by weight and height of children from birth to 5 years of age

Critical values	Interpretation	n (49)	%	95 % CI
**Weight/age**				
Z-Score < -3	Very low weight	0	0.0	0-7.2
-3 ≤ Z-Score < -2	Low weight	10	20.4	10.2-34.3
-2 ≤ Z-Score ≤ +2	Adequate weight	39	79.6	65.7-89.8
Z-Score >+2	High weight	0	0.0	0-7.2
**Stature/age**				
Z-Score < -3	Very short stature	3	6.1	12.8-16.8
-3 ≤ Z-Score -3 < -2	Short stature	6	12.2	4.6-24.8
Z-Score ≥ -2	Adequate stature	40	81.6	68.0-91.2
**Weight/Stature**				
Z-Score < -3	Marked thinness	3	6.1	12.8-16.8
-3 ≤ Z-Score ≤ -2	Thinness	6	12.2	4.6-24.8
-2 < Z-Score ≤ +1	Eutrophy	38	77.8	63.4-88.2
+1 < Z-Score ≤ +2	Risk of overweight	1	2.0	0.0-10.9
+2 < Z-Score ≤ +3	Overweight	1	2.0	0.0-10.9
Z-Score > +3	Obesity	0	0	0.0-7.2
**BMI/age**				
Z-Score < -3	Marked thinness	0	0.0	0.0-7.2
-3 ≤ Z-Score < -2	Thinness	4	8.1	2.2-19.6
-2 ≤ Z-Score ≤ +1	Eutrophy	40	81.6	67.9-91.2
+1 < Z-Score ≤ +2	Risk of overweight	3	6.1	1.2-16.8
+2 < Z-Score ≤ +3	Overweight	2	4.0	0.4-14.0
Z-Score > +3	Obesity	0	0.0	0-0.1

BMI: body mass index; CI: confidence interval.

SUPPLEMENTAL TABLE 2: Nutritional assessment by weight and height of children from five to 10 years old.

Critical values	Interpretation	n (13)	%	96%CI
**Weight/age**				
Z-Score < -3	Very low weight	0	0.0	0.0-24.7
-3 ≥ Z-Score ≤ -2	Low weight	2	15.3	1.9-45.4
-2 ≤ Z-Score ≤ +2	Adequate weight	11	84.6	54.5-98.1
Z-Score > +2	High weight	0	0.0	0.0-24.7
**Stature/age**				
Z-Score < -3	Very short stature	0	0.0	0.0-24.7
-3 ≤ Z-Score < -2	Short	4	30.7	9.1-61.4
≥ Z-Score -2	Adequate stature	9	69.2	38.6-90.9
**BMI/age**				
Z-Score < -3	Marked thinness	0	0.0	0.0-24.7
-3 ≤ Z-Score < -2	Thinness	0	0.0	0.0-24.7
-2 ≤ Z-Score ≤ +1	Eutrophy	12	92.3	64.0-99.8
+1 < Z-Score ≤ +2	Overweight	1	7.6	0.0-36.0
+2 < Z-Score ≤ +3	Obesity	0	0.0	0.0-24.7
Z-Score > +3	Severe obesity	0	0.0	0.0-24.7

BMI: body mass index; CI: confidence interval.

SUPPLEMENTAL TABLE 3: Nutritional assessment by weight and height of participants aged 10 to 20 years.

Critical values	Interpretation	n (12)	%	95% CI
**Stature/age**				
Z-Score <-3	Very short stature	0	0.0	0.0-26.5
-3 ≤ Z-Score < -2	Short	3	25.0	5.5-57.2
≥ Z-Score -2	Adequate stature	9	75.0	42.8-94.5
**BMI/age**				
Z-Score < -3	Marked thinness	0	0.0	0.0-26.5
-3 ≤ Z-Score < -2	Thinness	1	8.0	0.2-38.4
-2 ≤ Z-Score ≤ +1	Eutrophy	10	83.3	51.6-97.9
+1 < Z-Score ≤ +2	Overweight	1	8.0	0.2-38.4
+2 < Z-Score ≤ +3	Obesity	0	0.0	0.0-26.5
Z-Score > +3	Severe obesity	0	0.0	0.0-26.5

BMI: body mass index; CI: confidence interval.

SUPPLEMENTAL TABLE 4: Assessment of the nutritional status of adults ≥ 20 years of age by the Body Mass Index.

Age / Critical values	Interpretation	n (56)	%	95% CI
**20-60 years**				
BMI < 18.5	Low weight or malnourished	14	25.0	14.4-38.4
18.5 < BMI < 25.0	Adequate or Eutrophic	26	46.4	33.0-60.3
25.0 < BMI < 30.0	Overweight	13	23.2	13.0-36.4
BMI ≥ 30.0	Obesity	3	5.3	1.1-14.9
**≥ 20 years**				
BMI ≤ 22.0	Low weight	5	71.4	29.0-96.3
22.0 < BMI < 27.0	Adequate	1	14.2	0.3-57.9
BMI ≥ 27.0	Overweight	1	14.2	0.3-57.9

BMI: body mass index; CI: confidence interval.
