# Supplementary figures and images for: Nutritional status and vitamin A and zinc levels in patients with kala-azar in Piauí, Brazil
Source: Rev Soc Bras Med Trop. 2021 Sep 6;54:e0800-2020. doi: 10.1590/0037-8682-0800-2020 (PMC8437443; doi:10.1590/0037-8682-0800-2020)

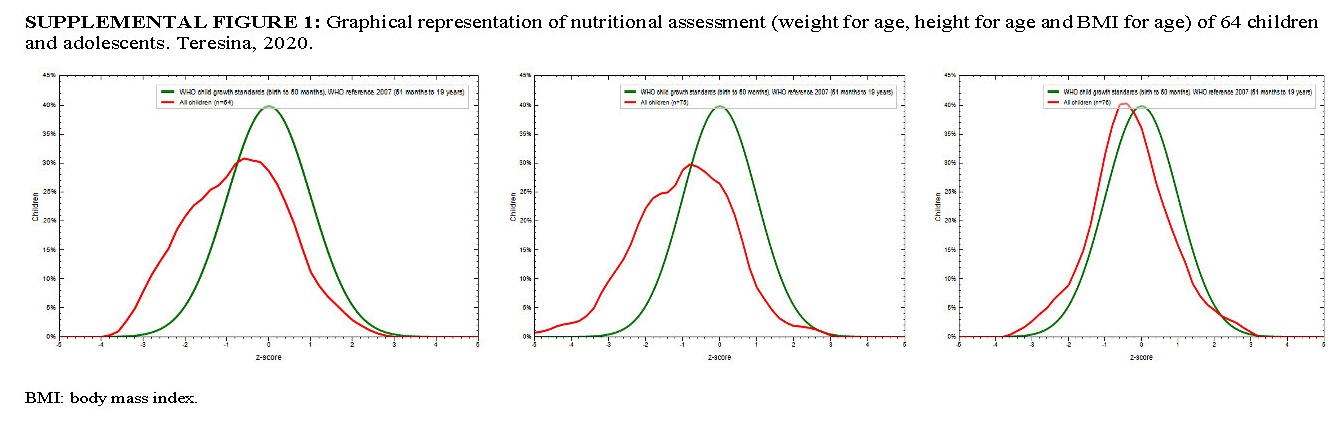

Supplement: Supplementary file 2 [file 1678-9849-rsbmt-54-e0800-2020-supp2.jpg]
