# Supplementary material for: Nutritional status and vitamin A and zinc levels in patients with kala-azar in Piauí, Brazil
Source: Rev Soc Bras Med Trop. 2021 Sep 6;54:e0800-2020. doi: 10.1590/0037-8682-0800-2020 (PMC8437443; doi:10.1590/0037-8682-0800-2020)
Supplement: Supplementary file 3 [file 1678-9849-rsbmt-54-e0800-2020-supp3.pdf]

SUPPLEMENTAL TABLE 5: Assessment of nutritional diagnosis with unified interpretation in different age groups by Body Mass Index.

Age (years)	Critical values	n	%	Unified interpretation
0-20	Z-Score ≤ -2	6	8.1	Thinness
	-2 < Z-Score ≤ +2	61	82.4	Eutrophy
	Z-Score > + 2	7	9.5	Overweight
20-60	BMI < 18.5	14	25.0	Thinness
	18.5 ≤ BMI < 25.0	26	46.4	Eutrophy
	BMI ≥ 25.0	16	28.6	Overweight
>60	BMI ≤ 22.0	3	75.0	Thinness
	22.0 < BMI < 27.0	1	25.0	Eutrophy
	BMI ≥ 27.0	0	0.0	Overweight
All ages	critical values for age	23	17.1	Thinness
		88	65.7	Eutrophy
		23	7.1	Overweight

BMI: body mass index;
